# A Cloud-Based Internet of Things Platform for Ambient Assisted Living

**DOI:** 10.3390/s140814070

**Published:** 2014-08-04

**Authors:** Javier Cubo, Adrián Nieto, Ernesto Pimentel

**Affiliations:** Universidad de Málaga, Departamento de Lenguajes y Ciencias de la Computación, Campus de Teatinos, 29071 Málaga, Spain; E-Mails: adrian@lcc.uma.es (A.N.); ernesto@lcc.uma.es (E.P.)

**Keywords:** ambient intelligence, ambient assisted living, Internet of Things, service-oriented, cloud computing, devices, sensors, services, gateway

## Abstract

A common feature of ambient intelligence is that many objects are inter-connected and act in unison, which is also a challenge in the Internet of Things. There has been a shift in research towards integrating both concepts, considering the Internet of Things as representing the future of computing and communications. However, the efficient combination and management of heterogeneous things or devices in the ambient intelligence domain is still a tedious task, and it presents crucial challenges. Therefore, to appropriately manage the inter-connection of diverse devices in these systems requires: (1) specifying and efficiently implementing the devices (e.g., as services); (2) handling and verifying their heterogeneity and composition; and (3) standardizing and managing their data, so as to tackle large numbers of systems together, avoiding standalone applications on local servers. To overcome these challenges, this paper proposes a platform to manage the integration and behavior-aware orchestration of heterogeneous devices as services, stored and accessed via the cloud, with the following contributions: (i) we describe a lightweight model to specify the behavior of devices, to determine the order of the sequence of exchanged messages during the composition of devices; (ii) we define a common architecture using a service-oriented standard environment, to integrate heterogeneous devices by means of their interfaces, via a gateway, and to orchestrate them according to their behavior; (iii) we design a framework based on cloud computing technology, connecting the gateway in charge of acquiring the data from the devices with a cloud platform, to remotely access and monitor the data at run-time and react to emergency situations; and (iv) we implement and generate a novel cloud-based IoT platform of behavior-aware devices as services for ambient intelligence systems, validating the whole approach in real scenarios related to a specific ambient assisted living application.

## 1. Introduction

Ambient intelligence (AmI) constitutes a generation of intelligence computing where a large variety of sensors and devices are present everywhere and for everyone at all times [1]. In the AmI domain, one of the main challenges is to produce software embedded in everyday objects and devices, in order to support different types of users and applications. An important aspect in AmI is the use of context-aware technologies, such as wireless sensor networks (WSNs) [2,3] to perceive stimuli from both the users and the environment. Nowadays, there are a wide variety of AmI systems [4], such as ambient assisted living ((AAL), http://www.aal-europe.eu), providing an ecosystem of medical sensors, computers, wireless networks and software applications for healthcare monitoring. Thus, AAL applications support the provision of user-dependent services for elderly and disabled people, such as monitoring the vital signs or controlling the movement tracking of a person, through the analysis of sensed heterogeneous information and detecting and reacting to problematic situations. Therefore, AmI systems have to deal with a wide variety of devices, ranging from smartphones and tablets, with medium capacity, to sensors, actuators, consumer electronics and wearable devices, with critical resource limitations. All of these devices may act in heterogeneous environments with regards to the network access types. Moreover, other factors more closely related to software can be identified, such as the high diversity of operating systems, as well as the different application programming interfaces (APIs) available for each operating system delivered for specific devices. Therefore, some of the main characteristics of these environments are the large number of heterogeneous devices, the diversity of communication technologies and the variety of application requirements and software.

The Internet of Things ((IoT), http://ec.europa.eu/digital-agenda/en/internet-things) is a technology and a market development based on the inter-connection of everyday life objects with each other, applications and database data. These devices, objects or things (such as laptops, smartphones, onboard computers, video systems, household appliances, intelligent buildings, wireless sensor networks, ambient devices, RFID tagged objects and commodities) are identifiable, readable, recognizable, addressable and even controllable via the Internet. This new Internet has led the evolution of the Ubiquitous Web 2.0, in integrating physical world entities into virtual world things, as some initiatives are already addressing (e.g., Xively (https://xively.com)). As pointed out by the European Research Cluster on the Internet of Things ((IERC), http://www.internet-of-things-research.eu/), things are expected to become active participants in business, information and social processes. The things may interconnect and communicate both between themselves and with the environment, by performing exchanges of data and information obtained from the sensing. Furthermore, they can react autonomously to real or physical world events and create services with or without direct human intervention. As the report considers, services could interact with these smart objects with standard interfaces, via the Internet, with the capacity of querying and retrieving information associated with the objects, considering issues, such as behavior, semantics or security. In this way, IoT will enable an ecosystem of smart applications and services, which will improve and simplify citizens' lives. The Future Internet has emerged as a new initiative to pave the way to a novel and dynamic global network infrastructure, with self-configuring capabilities, to meet the changing global needs of business and society. Therefore, future service-oriented Internet devices will offer their functionality via service-enabled interfaces adopting the vision of the Web of Things (WoT) (inspired by the IoT), e.g., via Simple Object Access Protocol (SOAP) based web services or RESTful APIs [5,6].

A common feature of Ambient Intelligence is that many objects are interconnected and act in unison, which is also a challenge in the Internet of Things. Although AmI is not part of the original concept of IoT, since it does not necessarily require Internet structures, there has been a shift in research towards integrating both concepts, IoT and AmI [7], considering IoT as representing the future of computing and communications, which brings us closer to Weiser's vision [1] of ubiquitous computing and ambient intelligence [8]. In fact, AmI's acceptability will come about through a balanced combination of operational technologies and embedded intelligence (EI) [9]. EI presents an artificial intelligence-oriented perspective of IoT, by revealing the individual behaviors, spatial contexts and also social patterns and urban dynamics with the capability of mining digital traces related to people during the interaction with smart objects [8].

However, the efficient combination and management of heterogeneous devices in the AmI domain is still a tedious task, and it presents crucial challenges. Therefore, there is a need to model and develop future Internet applications, such as AAL systems connected to the new Internet, supporting the interoperability between diverse stakeholders by governing the convergence between both the physical and the virtual worlds and by handling dynamic and continuous changes.

On the one hand, the IoT, including the mass of resource-constrained devices, could benefit from the web service architecture as today's web does. Recent work [10,11] has focused on applying the paradigm of service-oriented architecture (SOA) [12], in particular web services standards (SOAP, Web Services Description Language (WSDL), *etc.*) directly to devices. In general, applying SOA to networked systems is a crucial solution to achieve the reusability and interoperability of heterogeneous and distributed things. This would enable the direct orchestration of services running on devices with high-level services. For instance, sensors physically attached to an elderly person can offer, via web services, their context information or vital signs. Furthermore, these sensors could be easily integrated in a process that updates a feature (e.g., temperature) and location of the person, directly in the systems involved. Hence, the goal is to provide the functionality of each thing as a web service in an interoperable way that can be used by other entities, such as AAL applications in the ambient intelligence domain or other devices. However, adapting a given device to SOA is not a trivial problem.

On the other hand, in AmI systems, seamless mobile and fixed communication infrastructures, together with dynamic and massively distributed device networks, combined with natural-feeling human interfaces, offer independence for both device and user [13]. Similarly, cloud computing [14] promotes the seamless integration of digital and physical devices in the users' lives through device and location autonomy [15]. Therefore, combining both paradigms facilitates greater innovation and a new proactive computing approach, following the perspective of IoT, and new types of applications can be delivered more effectively and quickly [16–19].

In summary, in order to appropriately manage the inter-connection of diverse devices in AmI systems, we have to tackle different challenges: (1) to specify and efficiently implement the devices (e.g., as services); (2) to handle and verify the composition and interaction of devices coming from diverse sources; and (3) to standardize and manage their data for handling large numbers of systems together, avoiding standalone applications on local servers. In order to overcome the above challenges, in the work presented here, we propose a platform to manage the integration and behavior-aware orchestration of heterogeneous devices as services stored and accessed via the cloud. Three main aspects to generate the platform are completely introduced as new in this work: a common architecture using a service-oriented standard environment; a framework based on cloud computing technology; and the implementation of the cloud-based IoT platform. Furthermore, we have extended a previous model, as will be detailed in Section 3.2. Therefore, specifically, the main contributions of this work are as follows:

- We extend the description of a lightweight model for specifying the behavior of devices, to determine in a unified way (interface) the order of the sequence of exchanged messages (or operations) during the composition of devices, based on the concept of things or devices as a service.
- We define a common architecture using a service-oriented standard environment, to integrate heterogeneous devices by means of their interfaces via a gateway (in particular, devices based on the technologies of TinyOS, JavaME, IEEE 802.14.5 standard or Android) and orchestrate them according to their behaviors.
- We design a framework based on the cloud computing technology, connecting the gateway in charge of acquiring the data from the devices with a cloud platform (Google App Engine), to remotely access and monitor the data at run-time and react to emergency situations, which is a crucial issue for AmI systems in general, but especially for AAL applications.
- We implement and generate a novel cloud-based IoT platform of behavior-aware devices as services for AmI systems, validating the whole approach in real scenarios related to a concrete AAL application.

This paper is organized as follows. In Section 2, we motivate our proposal presenting the main problems to be solved and how they are addressed by our approach. Section 3 presents our proposal for the management and monitoring of devices as services showing the devices as cloud resources or services by means of a cloud platform, as well as the implementation of our platform. In Section 4, we describe the evaluation of the implemented platform. Section 5 analyzes some related work and compares it to our proposal. Finally, Section 6 outlines the conclusions and some future lines of research.

## 2. Overview of the Approach

In this section, we explain the motivation behind our proposal and the need for it, principally focusing on managing complex scenarios in AmI systems, such as AAL applications, in which heterogeneous devices are considered. We take advantage of the Internet of Things, service-oriented and cloud computing paradigms.

### 2.1. Problem Statement

According to the analysis of the European Commission [7], the demographic trends for Europe show an aging society with more people dependent on assistance. AmI and IoT may open up new possibilities for elderly and disabled people to live longer and safer at home and reduce the risks of errors in the dosage of drugs. Intelligent objects in the house could monitor and call for assistance if, for example, a person is still for an unreasonably long time in an unexpected location or situation or in the event that a fall is detected. Moreover, for health monitoring systems in hospitals, new platforms could be provided for the medical professionals to help monitor patients in emergency situations. In this sense, AAL has gained significance in recent years, combining aspects of intelligent platform design, assisted living solutions and ambient intelligence technologies in a coherent system comprising embedded sensors. Therefore, AAL applications will become a necessity due to demographic trends, a safe and robust design of smart systems being necessary, so as to control and ensure that the smart things really do serve both the patient by improving his/her quality of life and health professionals by helping them to quickly and automatically manage emergency situations.

#### 2.1.1. Challenges (1) and (2): Efficient Specification of Devices and Handling of the Composition of Heterogeneous Devices

**Figure 1.**
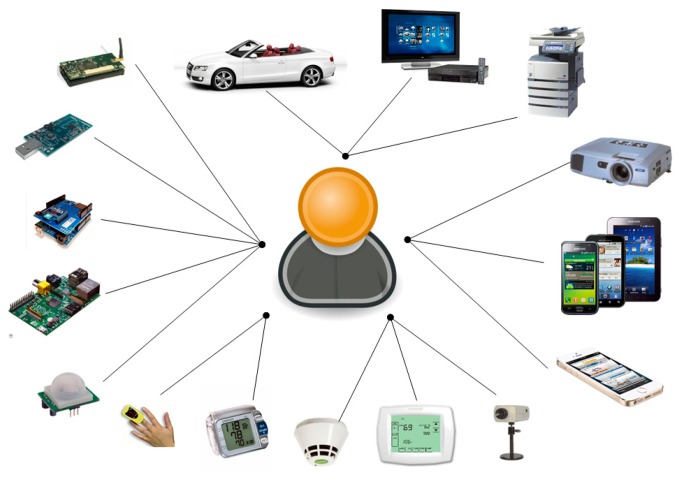
A heterogeneous system made up of sensors and devices.

An embedded WSN [20] in the healthcare domain consists of heterogeneous components and is used to monitor temperature, humidity, pressure, oxygen, carbon dioxide, heart rate, breathing rate, *etc*. Sensors and actuators-sensors are used to observe changing situations in the environment. Smart sensors or devices capable of sensing various dimensions in a smart environment are combined to develop a smart hospital, a smart home or any other environment to improve human life. In addition, a heterogeneous system, like an AAL system, may integrate diverse sensors or devices produced to different specifications, as shown in Figure 1. Therefore, it is necessary to design a common architecture to tackle the integration and communication problems, which could be addressed by considering a service-oriented device architecture (SODA) [21].

SOA standards were originally designed primarily to connect enterprise services. Therefore, the introduction of SOA to specify objects of the future Internet, such as sensor devices, brings with it new opportunities, but also new challenges. Real-world things are deployed on resource-constrained devices, e.g., with limited computing, energy and storage capabilities. Therefore, we need to study the simplification, optimization and adaptation of SOA standards to specify data and information received from these kinds of devices. Nevertheless, as even small, resource-constrained, networked devices get more and more powerful in peer-to-peer and pervasive computing applications, it is simply common sense to try to adopt the SOA paradigms in embedded device networks. Hence, several SOA initiatives, such as OSGi (http://www.osgi.org), UPnP (http://www.upnp.org) or Jini (http://java.sun.com/developer/technicalArticles/jini/JiniVision/jiniology.html), have evolved to interconnect heterogeneous devices and services. However, not all of them can equally adapt to others using the same hood. Furthermore, the lack of standardization makes programming for devices an arduous task. For this reason, a standard way for device manufacturers to develop devices for software developers and consumers is needed, while still providing developers with a standardized API.

In order to address this issue, the emergent OASIS (Open Standards for the Information Society) standard Devices Profile for Web Services (DPWS) [22] has been designed as a set of guidelines based on WS-* specifications to provide interoperability between different devices and services in a networked environment, e.g., a printer, a smartphone, a sensor or other new devices can detect DPWS-enabled devices in a network. Some convincing points in favor of DPWS are that it is an OASIS standard, it employs a web service mode built onto the standard W3C Web Service architecture and it has been natively integrated into the Microsoft Windows© operating system since Windows Vista (WSDAPI, http://msdn.microsoft.com/en-us/library/windows/desktop/aa826001(v=vs.85).aspx). In DPWS, each device is abstracted as a service where features of the device are exhibited as hosted services. The comparison between the important properties of reuse and research challenges of web services shows a gap in the use of DPWS in the future, centered on reusability [23]. DPWS shows that, for example topics, such as business processes, context dependencies or quality factors, have to receive more attention to increase the reuse of DPWS devices and to use this standard more easily in the field of software engineering. Therefore, for the development of future Internet service-oriented applications and the correct exploitation of the composition between things, it is crucial to define rigorous methodologies. These methodologies should not only consider features, such as signature, eventing mechanisms, security and discovery, which are currently considered in DPWS, but also complex real-world integration, such as those involving complicated business processes.

In order to fulfill this goal, in our previous work [24], the need to explicitly represent the (implicit) behaviors of things in order to develop applications in a more rigorous way was established. Specifically, that work promotes the use of WS-* technologies to specify service interfaces of things by extending the standard DPWS with behavioral descriptions. WS-* architectures are mainly used to take advantage of their capability to handle the interoperability among services considering complex business processes. As pointed out in [6], by default, although there is no notion of state in SOAP and the WS-* stack, the WS-Resource Framework (https://www.oasis-open.org/committees/tc_home. php?wg_abbrev=wsrf) may be used to cover the interaction with stateful web services, by managing the stateful resources of web service interfaces. The main purpose of this is to aid developers in the implementation of DPWS-compliant things (or devices) that host services by considering their behavior in terms of the order in which the actions, visible at the interface level, are performed. We consider that this challenge is vital to control the behavior of heterogeneous things during their compositions in the highly dynamic environments of the future Internet, as may be the case for AAL applications. These compositions will allow the creation of new applications generated as mashups of things where some concerns have to be handled, like, for example, the composition may violate the behavior of the devices (provoking lock situations) and some of their features may change at run-time. In addition, there is a need to address the integration of heterogeneous devices coming from diverse sources, which could be tackled by means of an intermediate piece and an appropriate language, so that all of the devices can connect in a unified way.

#### 2.1.2. Challenge (3): Seamless Management of Data in AmI Systems

In the AmI domain, a typical AAL application will generally consist of a target user, smart sensors, actuators, wireless networks, ubiquitous devices and underlying software services [16]. The data are initially processed and transmitted in a local point, and then, all of the sensors and devices of the system compose the collection of data and sensed information. The range of data to be monitored in these systems is highly variable in accordance with the diversity of these kinds of resource-constrained devices. Thus, in future AAL applications, a new sort of personal medical equipment will offer the possibility to place temporary sensors on/in the patient, providing located measurements of vital parameters. Taking advantage of this, the patient could stay at home with the necessary equipment, being able to connect to the hospital in the event some emergency situation occurs. This may replace costly trips to the hospital and reduce patient stress. Therefore, in order to maintain quality healthcare services, it is essential to have an intelligent, highly-resourced AAL system that is efficient, responsive and, most importantly, adequately ensures patient health. Therefore, in AAL applications, it is necessary to keep devices intercommunicated through a platform or framework (e.g., controlling emergency situations in such scenarios as home automation, remote healthcare monitoring systems or hospitals *in situ*). This could be performed via a cloud-based platform, which provides a seamless integration between the physical and virtual world devices and services.

A cloud-enabled platform eases the management of these systems, allowing simplified user access and effectively handling demand elasticity. The need for a large computing space and the ready availability of cloud services has led us to envision and design a real-time cloud-based framework. Therefore, the integration of cloud computing will allow the diversity of services for AAL systems to be expanded, since it enables all users (patients, caregivers and healthcare professionals) to acquire, gather, visualize and handle large amounts of data from diverse kinds of service providers and AAL systems. The immense processing power of cloud computing facilitates the highly quick processing of data and provides fast responses to the user environment.

### 2.2. Motivating Our Proposal

**Figure 2.**
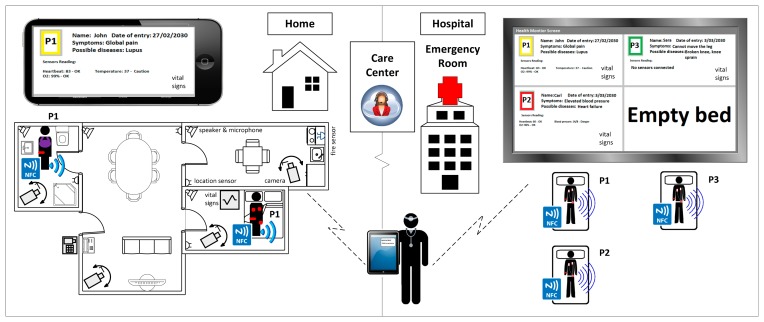
Emergency monitoring system: remote (home) and local monitoring (hospital).

In this section, we use a real-world example, an emergency monitoring system (EMS) (see Figure 2), to motivate and illustrate our approach, by demonstrating the need for a platform that integrates heterogeneous devices, as well as storing and managing the sensed and monitored information.

The main goal of this system is both to assist the patients and to help medical professionals in the seamless health monitoring of a large number of patients and data at run-time. The system is composed of sensors and devices connected to the patient, in order to detect and prevent any emergency situation, both in the hospital and at home.

Therefore, the end-users of our system are:

- The patients, who may be located in the hospital or at home. The different rooms of the hospital and also the monitored patient's home may hold a set of devices available as services and connected to the emergency monitoring system (via a local network or the Internet, respectively).
- The health professionals or specialists (typically a doctor or a nurse), who are usually found in their offices or the emergency rooms. Each specialist may access a set of devices available as services sending information related to the patients.
- The remote care centers, which are in charge of remotely helping the health professionals assist the patients being monitored, with intermediate processing of the information. The care centers may be managed from the hospital or externally, and they do not always need to be part of the system.

The system being considered is made up of the following components:

- Sensor nodes to measure basic vital signs of the patients, such as oxygen saturation or temperature.
- Devices with more complex behavior than a single sensor, used for sensing (e.g., a sphygmomanometer or existing mobile applications to measure the two values of the blood pressure, high and low) or for controlling some other actions of the patient (e.g., a video camera with a complex behavior or a user badge to control the movements and some vital signs at the same time). They may comprise several sensors integrated and interacting with each other (e.g., the user badge or a patient's bed, where several sensor nodes are connected, working together).
- A discovery application to find the existing sensors and devices in the environment to be connected and associated with the patients. Different profiles are considered to deal with the potential privacy issues (e.g., a patient can only view the devices sensing information related to himself, and a specific specialist can monitor all his/her patients).
- A dashboard application, used by the medical professionals or the care centers, to access all of the sensors and devices connected, as well as to manage the monitored information; which may be developed either as a mobile or desktop application for local monitoring (in the hospital, using a device connected to the local network) or as a cloud application with the capability of remote monitoring (at home or also in the hospital connecting to the cloud).
- A data repository hosted on a cloud platform to register the users and to store and manage the sensed and monitoring information from the system at any given moment; which contains the register of the user profile (patients, specialists and also the people working at the remote care centers), context, sensors/devices and services (inactive and active, *i.e.*, connected to a patient being monitored), is used as the central processing point for the requests and historical triggers of all data and events and is also responsible for maintaining a patient's schedule.

In our EMS system, two scenarios can be distinguished. First, a common scenario is when a new patient arrives at the hospital (right-hand side of Figure 2). Using the patient registration system (included in the data repository), the receptionist checks whether the patient is already registered in the system, in order to assign him/her a unique identifier by using a Near Field Communication (NFC) wristband. The patient is then transferred to the emergency room, and with the bracelet on, depending on the patient's urgent symptoms, a specialist decides which kind of sensors or devices he/she needs to be connected to. The emergency room is prepared with a gateway or an appropriate device to group all of the sensors, connected to the Internet or the hospital's intranet. The sensors are associated with the patient by means of the application's dashboard, using the NFC Universally Unique Identifier (UUID) and the patient's identifier number (ID_Number). In this way, the specialist may access the specific sensors associated with a specific patient and check his/her vital signs, as well as establish priorities of execution for the devices currently sensing.

**Figure 3.**
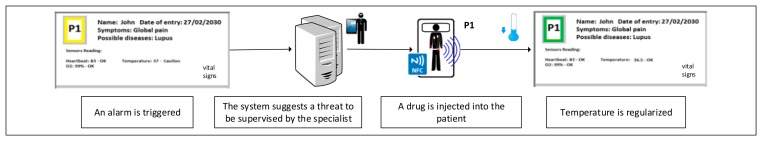
A sequence of actions performed by the EMS system in an emergency room (hospital).

Different options are possible to access the sensors. The first option, using a cloud dashboard application, allows monitoring of the data stored in the data cloud repository. This remote monitoring can, directly via the system or the care center, warn the doctor of emergency situations (e.g., oxygen saturation or temperature are not within normal values) or potential incompatibilities between drugs based on time restrictions (e.g., a specific pill every eight hours, incompatible with another specific drug) and help to provide the correct medication by avoiding possible human error. The second option, similar to the first, but using a mobile dashboard application, a tracking of the patient values may be performed locally from different places of the hospital or even with the possibility that the expert doctor may be in a different hospital (or building) from where the patient is, considering the sensors are running in a hospital intranet. Furthermore, as the third and worst case (managing the integration of devices, but not presenting advantages with respect to the current systems), the specialist could directly check the vital signs of the patient through the built-in screen in the sensors, as is done in most of the current health monitoring systems. Figure 3 depicts a possible situation in an emergency room.

In a second scenario, the previous situation may be also considered for tracking the patient's health, while he/she is at home (left-hand side of Figure 2). For example, a lot of oncology or cardiac patients, who require continuous monitoring, prefer to stay at home with their relatives more than staying in a hospital room. Their quality of life could be improved by providing them a home health gateway communicated with the hospital and care centers, gathering the sensed data via the cloud platform. This home gateway, in essence, provides the same functionality as the gateway installed at the hospital, but without enabling modifications of the sensor status or assignments, since in this modality, the sensors are usually used as read-only devices (they will not be able to be linked or unlinked to different devices, as occurs in the hospital). Therefore, in this scenario, when the patient leaves the hospital, the specialist sets up the sensors connected to his/her own home gateway. Once the patient arrives home, he/she connects the home gateway to the Internet, and forthwith, the specialist can perform practically the same as when in the hospital, since in this variety of the system, the cloud-based interface is used for remote monitoring.

Broadly, with the description of both scenarios, we demonstrate the need for specifying and handling the integration and composition of heterogeneous devices, as well as the seamless management of the sensed and monitored data in these kinds of real-time scenarios. Next, we illustrate our proposal with a more specific running example in the AAL area.

#### Running Example

Let us consider a concrete use case for the second scenario, remote monitoring at home, in which several vital signs of Alice, a cardiac patient, are periodically being monitored. In addition, due to Alice's heart problems, an accidental fall report mechanism in her smart home should be implemented. The goal in such a use case is to monitor Alice and, through the analysis of sensed information, detect situations that may be interpreted as a fall or abnormal values in the vital signs. Thus, if a fall is detected, actions are automatically triggered, such as issuing an alert to the care center or the monitoring of some of her vital signs (by means of sensors always connected to her). Some useful information needed to detect a fall is Alice's location, time spent in the same position, unexpected movements and body position. Therefore, the environment must be fitted with sensing units capable of capturing this information together with actuators able to contact the remote care center whenever a fall or strange movement is detected. Moreover, processing units are needed for analyzing the sensed data, including rule-based logic and image analyzer algorithms. There are several proposals for the applications of accidental fall reporting [25,26] with different degrees of complexity that provide results with different levels of accuracy to detect if the fast movement could be due to an accidental fall or a different action, such as sitting down (but this analysis is beyond the scope of this work) [27]. Therefore, in this specific use case, the system puts into action the following devices: sensors measuring vital signs (oxygen saturation, temperature, heart rate and breath rate); a sphygmomanometer measuring the blood pressure; a complex device to detect falls, named a user badge node (equipped with two three-axis accelerometers, circuitry for voice transmission over the radio and a sensor sensing the heart rate); and several video cameras and location sensors installed in different rooms of Alice's home; everything connected with the home gateway (even some microphones and speakers could be connected); all of these together with the corresponding devices at the care center, such as a media player device for video streaming, and at the hospital, to check on the patient.

**Figure 4.**
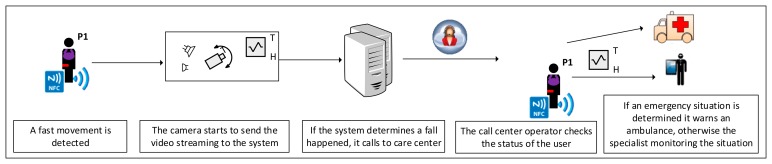
A sequence of actions performed by the EMS system in remote monitoring (home).

Figure 4 depicts a possible sequence of actions for this situation:

(1) When a fast movement is detected through the accelerometers of the user badge (the two accelerometers have to be executed at the same time), immediately the heart rate is checked (it must be checked periodically anyway) and a timer is triggered;
(2) The camera turns on and starts to send the video streaming to the system;
(3) In the case that it determines (by means of the corresponding analysis) that a fall may have happened and the patient does not cancel the alert procedure before the timer expires, then the voice transmission incorporated in the user badge is activated (just at this moment, as it makes no sense to activate it before this moment), and an automatic call is made to the care center;
(4) The person at the care center can talk to Alice and, at the same time, image processing of the streaming received is reproduced by the media player device (the operations on the cameras can be executed from the care center, such as moving the camera or zooming in), analyzing Alice's position to determine her status (in the case of accidents, it is important, for instance, to detect the position of the head);
(5) In the case that Alice is unconsciousness or unable to speak, receiving the automatic call is enough to alert the person at the care center to send help, otherwise, for example, Alice may explain that her situation is stable; then, the care center may simply alert the specialist about the situation to be managed remotely; and finally,
(6) In the control procedure, the specialist could ask Alice if she is able to connect all of the sensors to check the vital signs, and in this way detect what has caused the emergency situation. For instance, observing the heart rate and the blood pressure, it could be determined if chest pain (due to Alice's heart problems) provoked the fall, and in this case, an actuator would proceed to inject a dose of nitroglycerine every five minutes, with a maximum of four doses in case the heart rate does not decrease and the blood flow does not increase.

This particular situation necessitates many devices to be interconnected correctly, by means of an appropriate platform, with the corresponding mechanisms. Thus, all of the devices in Alice's home are connected either to the home gateway (the sensor nodes) or directly as services to the Internet (also the gateway is connected to the network), which communicates with a cloud platform managed from the care center and/or the hospital.

Therefore, this use case requires the handling of the behavior of the heterogeneous devices in action, not only to achieve a correct working of the interactions, but also to obtain the appropriate specifications of each behavior-aware device as a service and to store the monitored data.

Therefore, firstly, we model the devices as services by using the DPWS standard and specify the behavior of the devices, to determine the sequence of exchanged messages during their composition. To solve the heterogeneity, we use a service-oriented environment and a DPWS-compliant gateway, which will be used to orchestrate the devices according to their behavior.

Then, we design and implement a cloud-based IoT platform to remotely access and monitor the data at run-time, to react to emergency situations. The cloud platform makes the emergency monitoring faster, cheaper and a more reliable method of processing the information, since large, heterogeneous and complex data processing and visualization can be computationally intensive, especially for complex case studies. In the next section, we describe our proposal in more detail.

## 3. Cloud-Based IoT Platform for the Integration of Devices

This section presents our platform to manage the integration and behavior-aware orchestration of devices as services stored and accessed via the cloud.

Firstly, we introduce the service-oriented platform architecture to provide a better understanding of how it works. Secondly, we present how to specify devices as services, considering their behavior while they are being composed or orchestrated. Thirdly, the modeling of the Cloud of Things approach adopted by our proposal is described. Lastly, we give some details of the implementation of the platform, focusing on the domain of AmI systems, specifically in an AAL application. All of these points have been tackled with the intention of solving the problem of the integration of devices, not only thinking about a set of things connected, but also considering a general solution by analyzing, designing and implementing our final platform.

### 3.1. Platform Architecture to Manage the Heterogeneity of Devices

**Figure 5.**
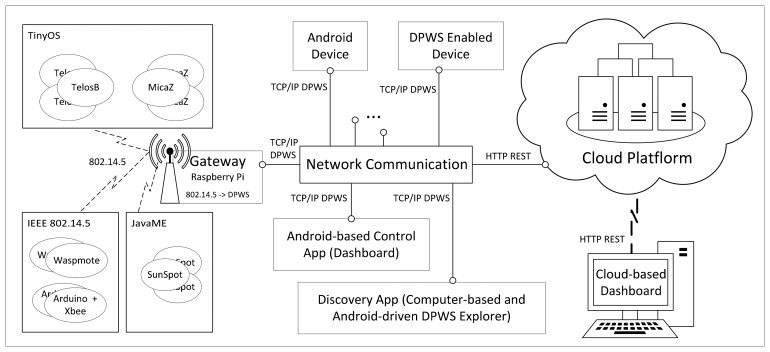
Architecture of DEEP (Devices Profile for Web Services (DPWS)-enabled devices platform).

In Figure 5, we depict the architecture of our platform, named DEEP (DPWS-enabled devices platform), whose main goal is to efficiently and seamlessly integrate and manage multiple and variable devices in diverse domains of human life, such as AAL systems.

We consider the following sensors, devices and applications to be integrated in the DEEP platform:

- Sensor nodes: TinyOS devices (TelosB and MicaZ sensors), JavaME devices (SunSpot sensors) and devices based on the IEEE 802.14.5 standard (such as Waspmote or Arduino + Xbee).
- Complex devices: Android devices, DPWS-enabled devices with a more complex behavior (such as a bed with many sensors connected acting as a complex device, a video camera or a printer) and Raspberry Pi used as the gateway related to the sensor nodes.
- Discovery and control applications: Discovery applications (Computer-based DPWS Explorer tool (http://ws4d.e-technik.uni-rostock.de/dpws-explorer/) and Android-driven tool (http://ws4d.e-technik.uni-rostock.de/tool-ws4d-droid-commander/)) and an Android-based control application (Dashboard), used to discover and manage all of the sensors and devices connected. This component is also used to orchestrate the interaction between the devices according to the scenario desired.

The platform principally consists of three components:

- The (sensor) gateway (at the level of sensors): it is used to transform the messages received from the diverse sensors (by means of the IEEE standard 802.14.5) to DPWS-compliant services (built over a TCP/IP stack), in order to provide a generic way (the concept of devices as services) to retrieve sensor data inside a local network. At this level, the sensor data have already been acquired and may be checked directly in the sensors themselves or in the local network. In addition, although, in principle, the DPWS standard requires that the devices are connected to the same network, the concept of the DPWS proxy enables devices hosted in other networks to be connected.
- The network communication (at the level of devices and applications connected directly to the Internet): it is in charge of communicating all of the heterogeneous devices (via TCP/IP), such as the sensors grouped through the (sensor) gateway, the more complex DPWS-enabled devices and the Android devices, as well as connecting (also using TCP/IP) the discovery and control applications (DPWS Explorer tools and Android dashboard).
- The cloud platform (at the level of data): it collects pushed information (data repository) from the network communication using a REST-based API, and its main function is to ease the remote management (storing, accessing and monitoring) of the sensed data at run-time, allowing simplified user access and effectively handling demand elasticity, reacting to certain situations according to the monitoring results.

In short, all sensors communicate via radio technology, more specifically by means of IEEE 802.15; all of them are concentrated in a (sensor) gateway, and the rest of the devices and also the gateway are connected via TCP/IP to the network communication. The network communication connects with the cloud platform, via HTTP REST, by taking the raw sensor values and gathering them, for instance, into the Google Cloud Platform, *i.e.*, Google App Engine. Thus, our system allows remote monitoring, by means of the push messages of the Google Cloud Messages or even thanks to BigQuery (Google large-scale data analytics) to suggest plausible diagnoses to the specialist. The next section presents the model proposed to specify the behavior of complex devices, which is considered while composing heterogeneous devices, illustrated in our running example.

### 3.2. Behavior-Aware Orchestrations of Devices as Services

**Figure 6.**
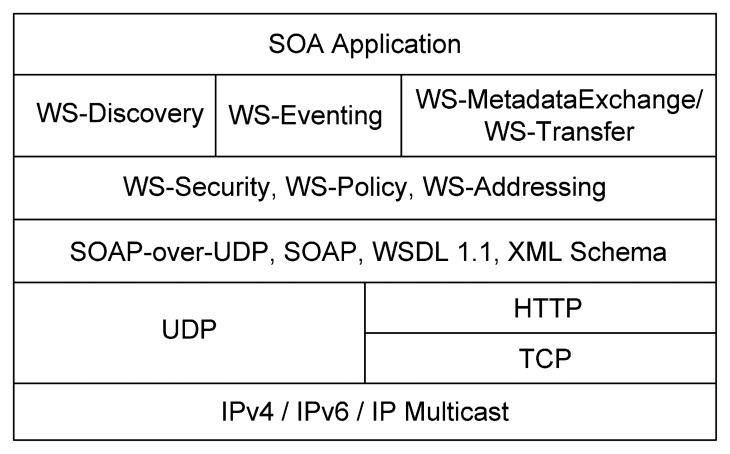
DPWS protocol stack.

As mentioned, we propose using the standard DPWS to describe devices as services. Thus, in DPWS, each device is abstracted as a service where features of the device are exhibited as web services (a device may be composed of several hosted services). DPWS uses the primitives of the web services architecture to create a framework for interoperable and standardized communication between embedded devices, as depicted in Figure 6. The key differentiator is that DPWS is language-independent. This means that all kinds of device services can be written using any programming language. The web services specification languages, on which DPWS is based, are the following: (i) WSDL for describing the messages each hosted service is capable of sending and receiving; (ii) SOAP for transporting all of the messages; (iii) WS-Addressing for advanced endpoint and message addressing; (iv) WS-Policy for policy exchange; (v) WS-Security for managing security; (vi) WS-Discovery and SOAP-over-UDP (User Datagram Protocol) for device discovery; (vii) WS-Transfer/WS-MetadataExchange for device and service description; and (viii) WS-Eventing for managing subscriptions for event channels. Although some aspects, such as the management of security issues, are not tackled in this work (out of scope), they could also be handled considering the DPWS protocol stack.

In [24], the necessity of extending DPWS was demonstrated to facilitate the implementation of a device (or thing) as a full service considering that its WSDL description should specify not only the signature, but also the behavior together with the order in which input and output actions are performed while the networked system interacts with its environment. Input actions model methods that can be called or the end of receiving messages from communication channels, as well as the return values from such calls. Output actions model method calls, message transmission via communication channels or exceptions that may occur during the method's execution. In order to include this extension in the DPWS profile, rigorous and lightweight methodologies are defined to develop things by promoting WS-* technologies, to specify service interfaces of things and to add the (implicit) behavior of things to the DPWS profile. This extended DPWS specification helps developers to implement DPWS-compliant things (or devices) that host services by taking into account their behavior (using constraints or finite state machines) in terms of the order in which actions, visible at the interface level, are performed while things are composed. The model for specifying behavior-aware devices as services is as follows:

- Constraints: When only a partial order of the behavior of things is required, we propose using four types of behavioral constraints (in [7], only three constraints were proposed; here, we incorporate the executeAll) to be added to the guideline (statements) described by DPWS:
  $$\begin{array}{c} \left\{bi\}\text{afterAll}\{ai\right\}\\ \left\{bi\}\text{afterSome}\{ai\right\}\\ \text{onlyOneOf}\{ai\}\\ \text{executeAll}\{ai\} \end{array}$$
  where {*bi*} and {*ai*} are the actions of a service hosted in a device. The afterAll constraint is used to specify that any action {*bi*} can be executed only after all of the actions {*ai*} have been previously executed. The afterSome constraint is less restrictive than afterAll, since any action {*bi*} can be executed when some other action {*ai*} has been executed. The onlyOneOf constraint means that only one of the set of actions {*ai*} can be executed in an interaction session. Finally, we extend the constraint model with the executeAll constraints that indicate that all of the actions {*ai*} have to be executed at the same time.
- Finite state machines: In those cases where it is necessary to specify not only the partial order, but also the ordered full-sequence in operations with the corresponding states' changes according to the execution of the messages, we propose using finite state machines (FSMs) [28] as a simple and user-friendly graphic solution to represent the complex relationships between messages.

#### Running Example

Coming back to our scenario, we illustrate the model for specifying and handling the behavior of these complex services hosted in the heterogeneous and distributed devices that compose the system. Note, basic sensor nodes only sensing a single value (such as those measuring vital signs), will also be described as a service, although it is not necessary to specify their simple behavior.

**Figure 7.**
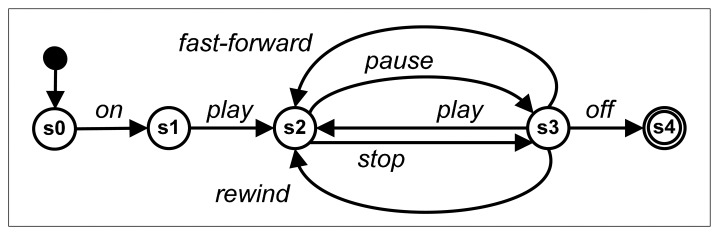
Finite state machine (FSM) for a media player device with a complex behavior.

- Constraints: For instance, the behavior of the user badge considers the communication between the two accelerometers, voice transmission and heart rate sensors, with operations, such as acc1, acc2, voice_on, voice_off or heart_rate. It can be specified as follows:
  $$\begin{array}{c} \text{C}1\_\text{b}:\;\text{executeAll}\;\{\text{acc}1,\;\text{acc}2\}\\ \text{C}2\_\text{b}:\;\{\text{voice}\_\text{on}\}\;\text{afterAll}\;\{\text{acc}1,\;\text{acc}2\}\\ \text{C}3\_\text{b}:\;\{\text{voice}\_\text{off}\}\;\text{afterAll}\;\{\text{voice}\_\text{on},\;\text{acc}1,\;\text{acc}2\}\\ \text{C}4\_\text{b}:\;\text{onlyOneOf}\;\{\text{voice}\_\text{off}\} \end{array}$$
  where constraint C1_b indicates both accelerometers in the user badge have to be executed at the same time to determine the movement of the user; constraint C2_b indicates that the voice transmission will only be turned on once the accelerometers have been executed (also a condition or interaction could be required to activate the voice, but at least, in the internal behavior of the user badge, the order is established like that); constraint C3_b means the operation to turn off the voice can only be executed after the operations to turn on the voice and to measure acceleration with both accelerometers; and constraint C4_b indicates that the deactivation of the voice, once it has been activated, will only be allowed once per iteration by ensuring, during the checking, that the voice will not be turned off. No constraint has been defined for the heart rate, since it is measured periodically without any restriction inside the device. As regards the behavior of a given camera (all of the cameras located in the home have the same behavior), the actions, such as move, record, zoom or halt, can be specified by means of the following constraints:
  $$\begin{array}{c} \text{C}1\_\text{c}:\;\{\text{zoom}\}\;\text{afterAll}\;\{\text{record}\}\\ \text{C}2\_\text{c}:\;\{\text{halt}\}\;\text{afterSome}\;\{\text{move},\text{record}\} \end{array}$$
  where constraint C1_c means the user has to authenticate before performing some of the actions related to moving, recording or zooming of the video camera; and constraint C2_c indicates that the operation halt to interrupt the recording can only be executed after some of the two operations, move or record.
- Finite state machines: The behavior of the service hosted in the media player (in the care center) requires a considerable number of exchanged messages (on, play, pause, stop, rewind, fast-forward and off) in a specific order, so its handling may require a more complicated model, such as the one provided by the FSMs. Figure 7 depicts the control of the full message sequence of this service using FSM representation.

The explicit specification of the behavior of things by means of constraints or full sequences with FSMs is the basis for developing behavior-aware compositions of things. These compositions will create applications generated in the form of mashups with new functionalities to be remotely accessed (e.g., as Software-as-a-Service—SaaS or Mashups-as-a-Service). For example, in our example, several devices are connected during the composition: oxygen saturation, temperature, heart rate, breath rate, location detectors, video cameras and media player; and some of them will trigger actuators or other devices (e.g., when the temperature is high, then the specialist is warned; or when a non-expected movement occurs, the heart rate is checked, a timer is triggered and the camera is turned on).

**Figure 8.**
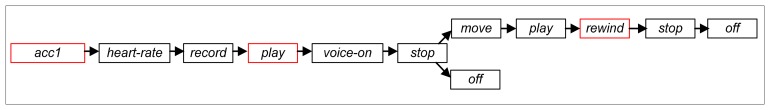
Specific orchestration (with violations) of the device composition for our EMS system.

The compositions by performing an orchestration of devices is specified by the dashboard control application of the platform presented in Figure 5. To do this, a process model of a mashup of things is proposed. This model is represented as a graph whose nodes represent single (only one action) or complex (more than one action executed at the same invocation) invocations (to actions of the devices) and whose edges represent a precedence relationship among these invocations (an edge from A to B states that Invocation B can be performed only after performing Invocation A). According to the example, different sequences of invocations can be executed in our scenario. Figure 8 presents a possible orchestration defined by the user (in our case, the orchestration could be defined by the healthcare professionals), where nodes represent the actions of each service hosted in the devices.

Although the verification of the behavioral composition is not a contribution of this paper, it is worth mentioning that it is required for checking whether or not a composition of things fulfills or violates the behavior, as proposed in previous approaches [24].

**Figure 9.**
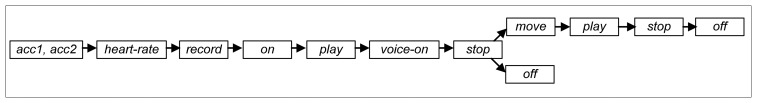
Correct specific orchestration of the device composition for our EMS system.

First, a simple and efficient verification technique at design-time is proposed. Therefore, in [24], a static verification technique was also defined, to check whether or not a mashup of things respects the behavior of the composed things specified at design-time, analyzing traces and actions executed from the orchestration specified by the user, according to a set of constraints and/or finite state machines, both determining the behavior of the things. Thus, a possible sequence of invocations of this composition could be acc1-heart_rate-play-voice_on-stop-move-play-rewind-stop-off, in which case: (1) acc1 is executed without acc2 at the same time, which violates the constraint C1_b specified for the user badge; (2) play will be executed with executing on, which violates the behavior defined in the automaton of the media player device (Figure 7); and (3) rewind could be executed without receiving immediately before one of the ordered sequences, play-stop or play-pause, which also violates the behavior of that service, since the first occurrence of play-stop is not considered, as play is executed again. Therefore, the static verification mechanism checks whether the composition respects the behavior of all devices (specified with constraints or FSMs) that are composed. Figure 9 shows the specific orchestration for solving the three violations detected by the algorithm.

Furthermore, a thing may change its behavior at run-time. Therefore, a change in the behavior of a thing may cause various compositions to no longer fulfill the behavior. Although compositions could be redesigned to comply with the new behavior, it would be appropriate to design run-time verification techniques to react when this situation occurs. Moreover, given that a thing can receive, at run-time, requests from instances of different mashups, these requests could violate the behavior of that thing, even though each mashup fulfills this behavior, because of the state's change of the thing. These kinds of situations cannot be detected at design-time, so run-time mechanisms are required to be aware of it and act accordingly. Therefore, it is a requirement to dynamically check and detect possible invalid invocations provoked by the behavior's changes. In [29], the static verification was extended with an approach based on mediation techniques and complex event processing (CEP) [30] to detect and inhibit invalid invocations, checking that things only receive requests compatible with their behavior. The proposal consists in processing invocations of services hosted in devices through a mediation platform, in order to detect and block the invalid ones using CEP techniques. The solution automatically generates the required elements for performing the run-time validation of invocations, and it may be easily extended to validate other issues, such as quality of service (QoS) and temporal restrictions.

### 3.3. Modeling the Cloud of Things in the AAL

Due to the need for a large computing space and the availability of services in AAL systems, we use a cloud platform offering a high-level abstraction, where services can be accessed easily via web service protocols. Therefore, this section describes the modeling of the Cloud of Things, the vision on which our approach is based. Specifically, we propose our solution as a cloud-based IoT platform for the fast integration and deployment of services over diverse kinds of sensors and devices. The platform centers on supporting applications based on the AmI paradigm, such as AAL applications, which are conscious of the location of the users and the environment state, as well as allowing them to be intercommunicated through the platform (emergency situations in scenarios of healthcare monitoring systems or hospitals). We, therefore use a cloud solution because it is centered on the user and offers an efficient, secure and elastically scalable way of providing and acquiring devices described as services. We take advantage of the processing power of cloud computing to easily process huge amounts of data coming from diverse devices, providing a fast response to the corresponding user. Furthermore, since traditional context management systems are incapable of handling large numbers of AAL applications together, our solution places the emphasis on handling a large number of users simultaneously. In this way, the context derived from one AAL system will become context knowledge for another AAL system inside the cloud repository. This is how the cloud solution will be an extendable knowledge source of context for AAL and will be able to deliver assistive actions quickly. In addition, the applications generated in the form of mashups with new functionalities will be displayed in the cloud. Mashups have surpassed the notions of integration and convergence and have become an important new trend that permeates all of society. Service mashups indicate a way to design and develop novel and modern web applications by combining existing resources utilizing content from diverse sources or devices and web APIs.

**Figure 10.**
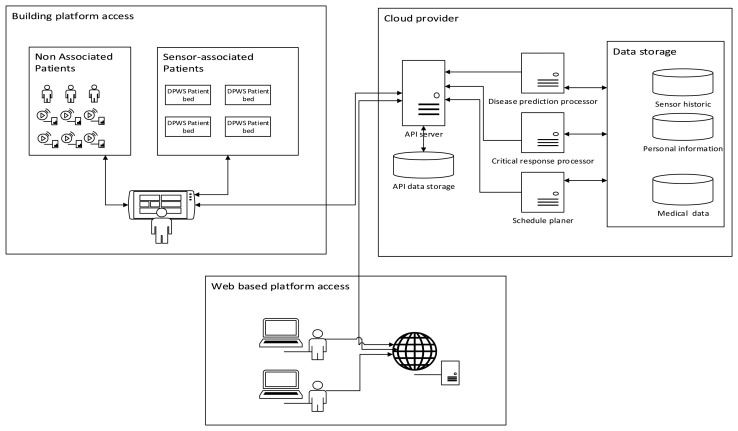
The Cloud of Things concept adopted for our platform.

Figure 10 shows how we model the Cloud of Things, which has been adopted for our platform, relating it to the AAL application described in our example. We use this concept of the Cloud of Things as a system to support decisions related to the processing of the connections among diverse things, managing the data and sensed information stored in the cloud and accessed as cloud services. Using both the storage resources and the capacity of the computation of cloud computing, large amounts of data and service information may be stored, analyzed and processed. Furthermore, the cloud computing solution enables different users and applications to share and reuse information by reducing the extra cost. The main computation is performed in the cloud architecture, so sensors and devices can handle other specialized processing tasks. Cloud services are easily deliverable to a system with an Internet connection. Therefore, our proposed solution actually simplifies the work of each component. It reduces the computing load of the sensors, helps disabled people and minimizes the work of healthcare professionals.

#### Running Example

With the purpose of obtaining a global vision of the DEEP platform in our AAL example, Figure 10 illustrates the connection between devices and sensors at the TCP/IP level, abstracting the details at a low level. Three parts are considered:

- Platform access: this part represents the repository of active patients, which distinguishes between patients being connected to a sensor (non-associated patients) and patients already connected to some kind of sensor (associated patients). Irrespective of the patient's location (in the hospital or at home), the associated patients interact with the platform in a similar way. A specialist connected to the intranet of the hospital and using DPWS-compliant software may update the state of the sensors as regards the patients, by changing the patients from active to non-active and *vice versa*, as well as monitoring the values provided by the sensors. Since DPWS uses an interface based on the WS-* standard, the information related to the devices is exposed in the internal network communication (the DPWS standard allows new devices to be known), by helping the healthcare professionals with the management of the system (monitoring, alerts, *etc.*). This selected organization increases the stability and security of the platform, since the basic operations are not performed by a remote server.
- Cloud provider: this infrastructure is used to store and process the data, as well as to guarantee remote access to the information. The cloud platform allows handling the monitoring information in a fast, simple and secure way. The security of the platform lies in the security of the service provider, itself, which enables us to focus on the development of the external API. Moreover, since the information is gathered in different datacenters, the security against the loss of data, replication and integrity is guaranteed. Taking advantage of the intrinsic power of the cloud, three dedicated components may be included in our platform: (1) a prediction processor to process the stored information by predicting some problems; (2) a critical response processor to generate responses alerting the doctor to emergency situations; and (3) a schedule planner to obtain quick conclusions. The API BigQuery (https://developers.google.com/bigquery/) of the Google Cloud Platform facilitates the processing of large amounts of data.
- Web-based platform access: this third component uses the external API published by the cloud platform to develop applications as a web interface for the management of personal data or remote monitoring of patients and even mobile clients to perform any operation available in the API (e.g., retrieving the information of a specific patient).

### 3.4. Implementation of the DEEP Platform

**Figure 11.**
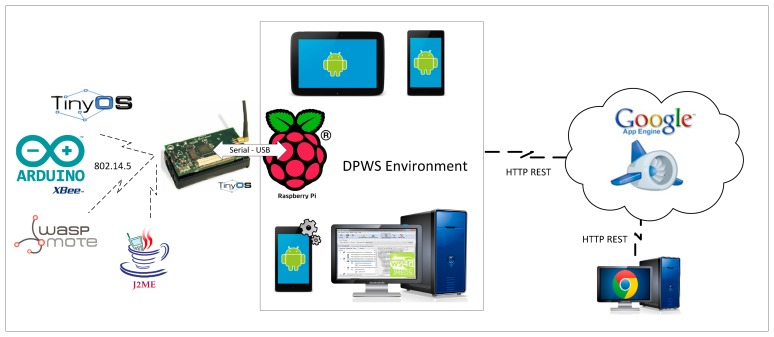
Technologies used for DEEP.

This section provides some details of the implementation of the DEEP platform, examining a prototype that has been developed (http://com-gisum-deep.appspot.com/). Figure 11 shows the technologies used for the DEEP platform.

Due to the heterogeneity present among the components of our platform and regarding the architecture previously depicted in Figure 5, three different abstraction levels can be distinguished: the (sensor) gateway, the network communication and the cloud platform.

- The gateway: the lowest level is represented by the gateway (Serial-USB) 802.14.5 for DPWS. This is executed on a low consumption board, Raspberry Pi. From the point of view of the implementation, the main functions of this level are as follows:
  ∘ Translate the data packets received from the sensors and devices externally connected by means of the IEEE 802.14.5 standard, with accessible information from a network TCP/IP. This information is displayed in the network with the DPWS standard, by establishing a generic way to access the information from any device connected to the network or even from devices connected to other networks by means of a discovery proxy (a functionality of the DPWS standard). The source code of the gateway is completely programmed in Java, except the communication libraries with the sensor motes, which are compiled with Java native interface (JNI) from the source code of TinyOS (open operating system for low-resources devices). These libraries can be compiled for the most common operating systems (Linux, Windows, OS X), which enables the deployment of the gateway in other, more powerful architecture, such as Intel MinnowBoard Max.
  ∘ Use the connection TCP/IP to connect to Google App Engine in order to provide the sensor data (e.g., values or associated patient), by means of an API REST, defined in the backend of the platform. The gateway tries to keep the information with the cloud provider for enabling the access to diverse queries externally to the intranet, safeguarding the security and privacy issues that could arise.
- DPWS environment: this second level is provided by the DPWS standard. It is presented as the alternative offline to control and monitor the sensors and is based on the DPWS standard, which provides an abstraction of the devices as services using the WS-* stack. With these standards, the devices have the capability to exchange the messages to be discovered and connected/disconnected to the system (hello, probe, metadata, subscribe, notification, *etc.*) by means of a search in the network according to the required and provided operations. A DPWS service proxy may be used as an aggregator of devices by extending the connection range to all of the networks to which the aggregator is connected. Thus, with an appropriate network configuration, whatever device connected to the intranet where the aggregated devices are deployed can be discovered and operated remotely.
- Cloud provider: this is the higher abstraction level. Google as the cloud provider has numerous functionalities for the backend with public APIs. Once the API has been created (the calls used, data types, *etc.*), it is possible to develop multi-platform applications accessing the data quickly and securely. Google provides tools for performing calls in a way that is transparent to the programmer for languages, such as Java, Python or PHP, which provides great flexibility for developing software. Focusing on our solution, we have developed a frontend to manage the patients' medical data, as well as monitoring in real time the record of the sensed values of the patients, saving infrastructure costs, as executing a browser would be possible with a limited computer, allowing access to the platform from a controlled environment.

#### Running Example

**Figure 12.**
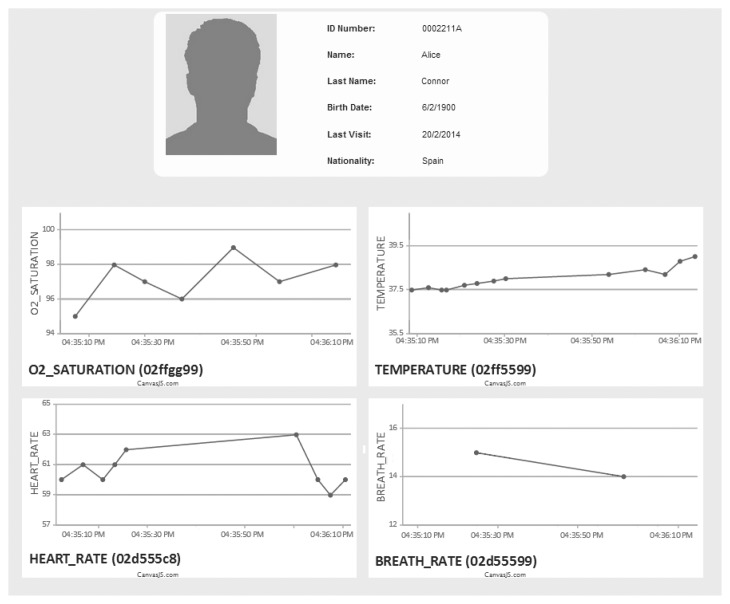
Screenshot of DEEP deployed on the App Engine cloud platform measuring the vital signs.

Coming back to our example, here we show the part of the cloud provider of our platform. Specifically, Figure 12 depicts a screenshot of DEEP deployed on the Google App Engine cloud platform, where the sensed and monitored information corresponding to the vital signs of the patient, Alice, in real time, can be seen (in the platform, this kind of information can be accessed, although sensors and devices need to be connected at the right moment to obtain the values in real time; otherwise the values already stored, not the real time values, are shown (http://com-gisum-deep.appspot.com/sensor_popup.html?patientID=0002211A&nfcID=e9957675-62e4-487b-bd88-1a077346450a)).

## 4. Evaluation of the Platform

In this section, we evaluate our implemented platform; concentrating on the usability and the advantages of the cloud platform used.

### 4.1. Usability

We have built our platform to provide an easy user interaction experience. We use a simple GUI, without unnecessary component and animations that may disturb the user. From the end-users of our platform (patients, health professionals or specialists and remote care centers), patients are really connected to the system; and, they do not need to manage the platform, but only to connect or disconnect some device, and the two latter ones will be the main users of the platform. To measure the time that it takes to use the platform, we have conducted a usability test measuring the time users need to perform two concrete use cases:

- Registering a new patient and linking to an NFC ID and, then, reverting the changes.
- Checking the vital signs of five patients.

We have conducted the usability tests with a group of 40 persons with different computer skills (working with computers, power user and web surfing and gaming) emulating the real end-users. We have requested users to start both tests using a computer without loading the website of the platform. In order to check the experience and learning, we have measured the time needed to accomplish both use case tests twice.

**Figure 13.**
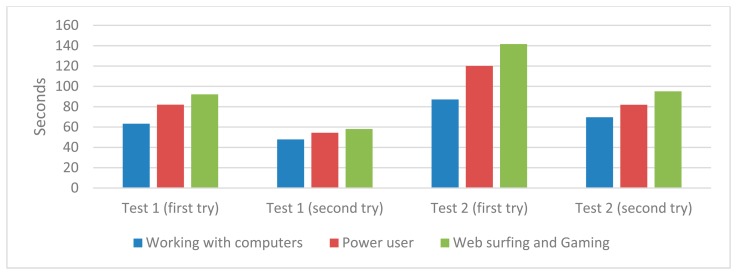
The time to perform two usability tests according to the user experience.

In Figure 13 is shown the time to perform each usability test according to the user experience. We observe that in all of the cases, the user (independent of her/his computer skills) learns to interact with the platform with a single try, decreasing the time for the second execution by 30% overall.

### 4.2. Scalability, Elasticity, Latency and Cost

Two main computing models have been dominating information technology for a while now: on the one hand, the centralized computing model, typical in mainframe systems with multiple terminals connected to them; on the other hand, the distributed computing model, with the client-server model, is the most widespread example. Recently, a new model, cloud computing, has emerged with the aim of providing support to the explosive growth of the number of devices connected to the Internet and complementing the increasing presence of technology in people's daily lives. Cloud computing provides economic, scalable and robust services over the Internet. Therefore, our platform benefits from these advantages of the cloud, which is evaluated as regards scalability, elasticity, latency and cost.

#### 4.2.1. Scalability

The scalability of the platform relies on the amount of data to be processed by the gateway. The most common interaction with the gateway is to receive new data from the sensors. We consider that a packet contains all of the relevant information about the sensors, whose overall size is around 16 bytes. The amount of sensors that a gateway can handle depends on how often we want to update the data and the technology used for the communication with such a sensor. In our scenario, we are using TinyOS-based motes (MicaZ and Telos B) connected with the Raspberry Pi through a serial interface. This kind of devices has a limitation as regards the communication with the computer. Considering an update rate of one packet per second, the receiver interface could handle theoretically up to 900 sensors (Maximum number of sensors (in theory) = 115,200 (bps) /8 (bytes) /16 (bytes/sensor) × 1 packet/s = 900 sensors) in the case of using TelosB transferring to 115,200 bps (MicaZ has a transfer rate of 57,600 bps). In order to connect more devices to our platform DEEP, we could upgrade the receiver hardware or simply add more gateways to the network.

However, the range of point-to-point communication inside IEEE 802.15.4 is between 10 and 100 m (depending on the throughput), which is clearly not enough to cover scenarios considering big buildings (like an emergency center) with a single gateway. Although in a real scenario, we could have around 50–100 sensors, in order to check the scalability of our gateway, we have performed a benchmark with a range from 30 to 3000 sensors, by simulating different sizes of emergency rooms.

The test consists of retrieving as much patients as it is needed to reach the desired amount of sensors. To avoid the IEEE 802.15.4 bottleneck, sensors have been generated in a simulated way. After adding the simulated sensors to the gateway, they immediately start to generate new data and notify the gateway about the generation. During the next 30 seconds, the gateway processes the new information and uploads it to the cloud platform. For all of the test cases we have executed, we have to wait until the cloud platform cools-down (to avoid active instances), in order to check how it adapts to the workload.

**Figure 14.**
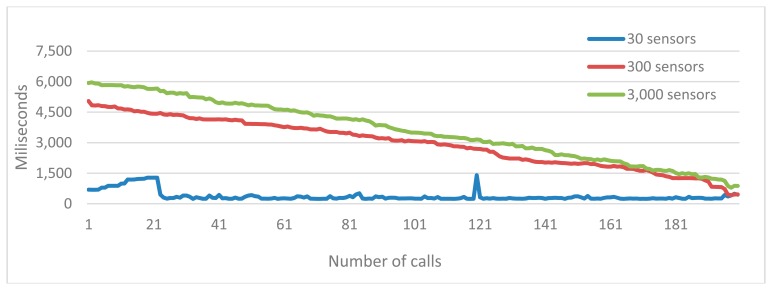
Platform average response with a single gateway.

Figure 14 depicts two relevant issues. The first one is how the cloud platform handles the load, in such a way if the load is big enough (more than 300 sensors per second updating), the cloud will begin an adaptation process until the response to be stabilized is as low as the smallest test (30 sensors). In our use case, it took around 30 s to be stabilized. The second issue is how a single gateway can handle more than 3000 sensors, since after the adaptation of the cloud platform, the average response time decreases until almost the response time of the smallest test size. Therefore, if in the future, we find a way to improve the hardware resources (more bandwidth, more range, *etc.*) to connect sensors to the gateway, then Raspberry Pi could deal with an enormous amount of sensors (overcoming the limitation of the IEEE 802.15.4 standard of 900 sensors).

#### 4.2.2. Elasticity

Elasticity is not exclusive to cloud computing, but it is easier to get elastic architectures via the tools provided by the cloud, especially through the virtualization and pay-per-use. In order to develop an “elastic” gateway solution, we should start and shutdown gateways on-demand (similar to the virtual machine solutions), depending on the workload. However, although this solution is not possible for the gateway, we could directly increase the hardware technology of the gateway used, *i.e.*, we can scale up and out the gateway infrastructure, allowing us to adapt our solution according to the size of the scenario.

Inside the cloud platform, there exists a degree of elasticity inside the architecture of servers for the hosting of web applications. Most typical is that the first layer (web servers) is elastic, while the database layer is simply scalable. However, in the Google App Engine, there is a highly replicated architecture, named high replication datastore (HRD) (https://developers.google.com/appengine/docs/java/datastore/)/(https://developers.google.com/appengine/docs/python/datastore/), which uses the Paxos algorithm (http://en.wikipedia.org/wiki/Paxos_(computer_science)) for the replication of data around the world, obtaining a solution that is totally elastic, both frontend and backend, that adapt themselves to the load, which is completely transparent to the user (see Figure 14).

#### 4.2.3. Latency

The latency of our solution depends on two main factors, the gateway and the cloud platform status. The gateway latency, as we explained previously in Figure 14, depends on how many sensors the gateway has to handle. However, in the running example in this paper, the computing capabilities of a Raspberry Pi seems to be enough to deal with three-times more sensors than the hardware involved in the communication can provide.

On the other hand, the latency of the cloud platform is practically independent of the size of the managed data, since the automatic scaling provided by the Google App Engine allows the capacity of computation to be increased, in moments when the traffic is intense, by activating instances (https://developers.google.com/appengine/docs/adminconsole/performancesettings#minimum) (as they are known in the Google Cloud) and also allows reducing the economic cost in case too much power is not necessary when there is not too much traffic in the platform (it is even possible to deactivate the machines).

It is worth noting that if it is searched, the scaling process is transparent to the user; another concept of instance is considered, inactive or idle instance, where Google reserves the instances to be paid in such a way that, if the application needs to be scaled, the code is already loaded and ready to serve traffic. Otherwise, some delays (of up to two seconds in our case) can be observed, which may become critical depending on the environment to which the platform is dedicated. In addition, the maximum latency for a response may be configured using the application's dashboard, ranging from 10 ms to 15 s.

#### 4.2.4. Cost

In order to estimate the cost, we use the table of prices per operations from the Google App Engine, where the cost of the manipulation of each entity is estimated (https://developers.google.com/appengine/pricing) (considering the fails to cache; otherwise, it could be considered as cost zero; otherwise, only the traffic cost would be considered, which is not being studied, due to the large volume of data being handled (https://developers.google.com/appengine/docs/adminconsole/memcache)). Table 1 presents a relation of cost estimation for a set of operations to be executed in DEEP, as regards our running example.

Furthermore, to these costs we should add the technical support, if it is needed, for the deployment and additional maintenance, such as phone support, which is quantified in a different way (https://cloud.google.com/support/).

To estimate the costs in a monthly average of operations, we assume that the registers have already been created, both for sensors and patients. Therefore, we only compute the costs of the active patients and the updating of the sensors. This is because the register/unregister processes are not very frequent compared to the data traffic generated by the query and the modification of the sensors.

Tables 2 and 3 give the results of this estimation for active patients and updated sensors, respectively. The traffic cost for a regular user depends on the operations performed on the platform (we consider that the cache is active; otherwise, the costs are very high).

In order to calculate the costs of the platform, we consider a user of the platform, e.g., a medical specialist with a common behavior pattern on his/her computer for one month, being able to perform the following actions:

- To visualize 300 lists of patients per day in one month (300 patients × 30 days = 9000 operations as a result of listing patients during one month).
- From the 300 listed patients, only the patients with sensors connected are monitored, let us say 100 patients being monitored for 15 s per day (100 patients × 15 seconds × 1 data refresh per second × 30 days = 45,000 operations of listing sensors).
- The rest of the values in the table do not follow a fixed rule; they depend on parameters, such as the number of sensors connected, times of accessing sections of the platform or even the interest the doctor has in a specific patient. These values have been obtained experimentally, and rounded up, to analyze the platform in a realistic scenario.

In order to determine an approximate cost according to three different kinds of hospitals managing data (depending on the number of supported patients), small hospital, regional hospital and network of hospitals, the previous data are cross matched to obtain the values presented in Table 4.

**Figure 15.**
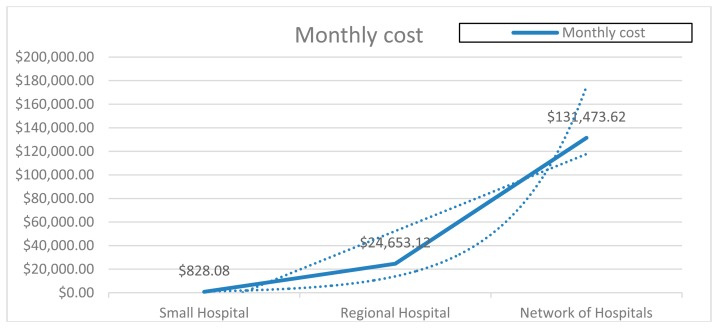
Monthly cost for different kinds of hospitals managing data with DEEP.

Figure 15 corresponds to the values in Table 4 represented graphically. On the one hand, it shows how the costs increase proportionally with the size of the hospital, which demonstrates that our platform is a good alternative for the use of the *ad hoc* infrastructure developed by each hospital. On the other hand, it does not consider the cost of the effort of migrating to this new technology nor the risk of the well-known vendor lock-in problem in the cloud paradigm, although some initiatives are already trying to address this problem.

## 5. Related Work

In this section, we analyze different approaches, focusing on the three main challenges addressed in this approach: specification and implementation of devices as services, handling of the composition of heterogeneous devices and seamless management of data in ambient intelligence, specifically in ambient assisted living.

With respect to the two first lines of research, recent approaches have made progress in displaying devices as services and handling their composition. In [31], both the state-of-the-art and the key research directions are identified, related to service-oriented middleware for the future Internet: service description, discovery, access and composition. Until recently, these aspects were only considered as services, but the future Internet is already a reality, so the necessity of considering contents, devices, sensors and things has to be included in any new challenge. As regards the choice of the most appropriate technology for specifying devices, some [5,6] have compared REST *vs.* WS-* technologies. They claim that RESTful web services are easy to learn and suitable for programming IoT applications, and their main advantages are their universality and the uniform service interface. However, they also argue that REST addresses only basic distributed interaction and coordination, leaving many open complex issues that have been tackled by WS-* technologies, such as dealing with service behavior, semantics or quality of service. In addition, WS-* specifications benefit from having a clearer standardization process than REST.

The SIRENA (http://www.sirena-itea.org/) European project has played a pioneering role, by applying the SOA paradigm to communications and interworking between components at the device level, with the main objective being to develop a Service Infrastructure for Real-time Embedded Networked Applications. The SIRENA results [32] were used as the foundation for both, the SODA (https://itea3.org/project/SODA.html) and SOCRADES (http://www.socrades.eu/) projects, with selecting DPWS as the best choice for achieving device integration in heterogeneous domains. Additionally, stemming from the SIRENA project, the initiative “Web Services for Devices” (WS4D) (http://ws4d.e-technik.uni-rostock.de/about/) also complies with the DPWS. WS4D brings SOA and web services technology to diverse application domains, such as home or industrial automation, automotive systems and telecommunication technology, by facilitating the setup and management of network connected devices in distributed embedded systems.

The ongoing FET (Future and Emerging Technologies) European project, CONNECT (https://www.connect-forever.eu/), drops interoperability barriers by synthesizing on the fly the connectors via which networked systems communicate [33]. It assumes that a networked system comes together with a labeled transition system (LTS)-based specification of its interaction protocol by specifying its behavior. Furthermore, in [34,35], the authors working on CONNECT propose deriving from the WSDL of a web service a partial ordering relationship between the invocations of the different WSDL operations, which they represent as an automaton, which models the interaction protocol that a client has to follow in order to correctly interact with the web service. The behavior protocol is obtained through synthesis (driven by data type analysis, obtaining a preliminary dependencies automaton and optimized by means of heuristics) and testing stages (to verify conformance). Compared to our approach, the CONNECT project first assumes that the behavior protocol has already been specified by LTSs, while we are proposing to specify service interfaces of things by adding a set of single constraints to the DPWS guidelines in order to determine the links in the interactions. However, even later, when they propose deriving a partial order of the message sequence of a service, their approach is too complex, and it does not easily maintain the compromise between the expressiveness and the scalability issues in a world composed of billions of resource-constrained devices, since both synthesis and testing processes are required. However, our approach is to apply rigorous and lightweight methodologies to develop things, by aiding developers in the implementation of DPWS-compliant devices.

Other efforts principally focus on the WoT vision, by both: (i) specifying, discovering and integrating things by means of a semantic web-based architecture [16,36,37]; and (ii) generating mashups of heterogeneous things [38,39]. However, the main gap in these proposals is that devices with a more complex behavior cannot be connected by using their mechanisms, since the implicit behaviors of things are not considered. Therefore, violations of the (implicit) behavior of things might happen, and the system could get locked out during its execution, for instance due to a deadlock situation. Our approach proposes designing the foundation on an extension of the DPWS profile by allowing the incorporation of behavior-aware things in the mashups generated without human intervention as regards the interaction protocol at run time, ensuring the composition works correctly. ThingML [40] is a domain-specific modeling language for efficiently providing communicating services on resource-constrained devices. The proposed language allows interaction protocols to be specified via state machines, and it would be worth investigating whether and how those state machines can be mapped into our FSMs, so as to allow their inclusion in WSDL interfaces as considered by DPWS.

There are other initiatives, such as the Open Services Gateway initiative (OSGi) (http://www.osgi.org/) and the Sensor Model Language (SensorML) (http://www.opengeospatial.org/ standards/sensorml). Specifically, the objective of OSGi is to create intermediate software for intelligent devices by facilitating their heterogeneity. Some approaches have used OSGi specifications to define a standardized, component-oriented, computing environment for networked services, for example, in systems for dependent people [41], although no effort has been made as to the efficient management of the large amounts of motorized data. The main objective of SensorML is to enable interoperability, first at the syntactic level and later at the semantic level. To do this, SensorML uses ontologies and semantic mediation; thus, sensors and processes may be easily shared among the intelligent sensor web nodes, used automatically in complex workflows and understood by machines. This standard is the result of an implementation standard of the OGC's Sensor Web Enablement (SWE) activity, which allows the integration and analysis of streams of sensor data coming from heterogeneous sensors and devices based on standards and managing the interoperability. Although here, in our first attempt, we opt for DPWS, because of the simplicity of this standard, as well as the advantage of the service-oriented architecture model for specifying more complex behaviors, we are also studying these other commonly used initiatives.

Regarding the seamlessness of the large amount of data being sensed in some real scenarios, such as in AAL applications, several proposals are currently addressing it and proposing solutions.

CloudIO [17] focuses on addressing the interoperability, accessibility and usability of systems based on WSNs through a platform based on cloud computing services. The developed platform is modular, flexible and scalable and integrates telemonitoring, personal communication and interactive services managed from a cloud infrastructure.

IAServ [18] (Intelligent Aging-in-place Home-care Web Services Platform) provides a personalized healthcare service ubiquitously in a cloud computing setting to support a desirable and cost-effective method of caring for the aged/aging in their homes.

CoCaMAAL [19] (Cloud Oriented Context-Aware Middleware in Ambient Assisted Living) presents a generic architecture to support AAL environments integrated in body sensor networks with context-aware service management systems using cloud infrastructure. These proposals exploit and benefit from the cloud in a similar way to our proposal; however, two main aspects addressed in our work are not supported in these approaches: the possibility of specifying the behavior of the devices, generating complex orchestrations and workflows of things, verified to check whether some violations may occur in the composition; and the integration of new devices in our DPWS environment, directly connected to the cloud platform used for storing the data received from DPW-enabled devices. Therefore, our behavior-aware model enables the healthcare professionals to specify diverse and complex orchestrations, depending on the scenarios that have to be controlled and monitored.

In [42], the authors propose a new AAL cloud computing approach based on ROS's messaging system, a cloud enabled robot operating system. One disadvantage of ROS is that publish and subscribe are anonymous, meaning that it has to be the overall system that guarantees data safety and confidentiality. To the contrary, our proposal integrates many heterogeneous devices, using the DPWS standard, which increases the stability and security of the platform. In addition, thanks to the cloud infrastructure, the responsibility for security lies with the service provider itself. This enables us to concentrate on the development of the external API.

Moreover, [43] presents a review of a variety of AAL applications, which are gaining in prominence and dominance in smart homes, the world over. Some approaches have proposed service-oriented solutions related to home automation systems, *i.e.*, for a home network system [44] and for a smart home [45]. The former presents a sensor mashup platform, which allows the dynamic composition of the existing sensor services. They mainly focus on helping non-expert developers to create context-aware services within the home network system, but their framework does not offer any guidance for controlling the behavior of the system, only messages that are exchanged by using WSDL and REST/SOAP. The latter is closer to our proposal. The authors propose an application logic distribution where devices in a smart home incorporate a set of rules that can govern their behavior, following ECA (event-condition-action) rules: they listen to external messages (notifications received from other services), and according to some conditions defined in these rules, they decide to perform their own actions. In comparison to our approach, this mechanism is not lightweight, and it can introduce too much complexity when dealing with resource-constraint devices, making it necessary to have a rule engine to analyze the rules. In addition, a rule is not enough to determine the correct order of operations of a service, since a full rule requires events coming from other services to be triggered. Therefore, the protocol detailing the partial order or the full sequence among operations (of a single device) cannot be generated in a simple way by means of ECA rules.

## 6. Conclusions

In this paper, we have presented a platform to manage the integration and behavior-aware orchestration of devices as services stored and accessed via the cloud in AAL applications. We have described our proposal of modeling the heterogeneous devices as services by using the DPWS standard (which has already been extended with the behavior specification of devices, with the purpose of determining the order of the sequence of exchanged messages during their composition). To solve the variability of the devices, we used a service-oriented environment and a DPWS-compliant gateway used to orchestrate the devices according their behavior. We have detailed the design and implementation of our cloud-based IoT platform for remotely accessing and monitoring the data at run-time, reacting to emergency situations, which is such a crucial issue in AmI systems.

We have validated the whole approach in real scenarios related to a specific AAL application, an emergency monitoring system, to help medical professionals with the seamless health monitoring of a large number of patients and data at run-time, by means of sensors and devices connected to the patient, so as to detect and prevent any emergency situation, both in the hospital (local) and at home (remote). We have demonstrated that the cloud solution eases the management of these systems, allowing simplified user access and effectively handling demand elasticity.

Currently, we are working on developing the historical triggers and the patient schedule. We are also refining the connection of the system with the appropriate actuators, in order to automatize as many parts of our solution as possible. As regards future work, we plan to evaluate and validate the whole solution from the very beginning of abstracting the devices as services through to the analysis of the monitored information by using the cloud.

## Figures and Tables

**Table 1.** Cost estimation of operations to be performed in DEEP.

Operation	Read Count	Write Count	SM Count	Cost per Operation ($)	Cost per Each 100k ($)
Register patient	1	2	1	2.50000E-06	0.25
Remove patient	0	2	0	1.80000E-06	0.18
Link patient with NFC	1	4	3	3.60000E-06	0.36
Unlink patient from NFC	1	4	1	3.60000E-06	0.36
Register sensor	1	2	1	2.50000E-06	0.25
Remove sensor	1	2	0	2.40000E-06	0.24
Update value of sensor	0	1	1	1.90000E-06	0.19
List patients	1	0	0	1.20000E-06	0.12
List sensors	1	0	0	6.00000E-07	0.06
Recovery patient data	1	0	1	7.00000E-07	0.07
Query of the last value of a sensor	1	0	0	6.00000E-07	0.06
Recovery all values of a sensor	2	0	0	1.20000E-06	0.12

**Table 2.** Cost estimation of active patients.

Operation per User	Average Monthly Operations
List patients	9000
List sensors	45,000
Recovery all values of a sensor (10% fails to cache)	225,000
Query of the last value of a sensor (8 hours/day, 5% fails to cache)	864,000
Recovery patient data	900,000

**Table 3.** Cost estimation of updating sensors.

Operation per Sensor per Second	Average Monthly Operations
Update value of sensor	864,000

**Table 4.** Operations and monthly cost for different kinds of hospitals managing data with DEEP.

Operations	Small Hospital	Regional Hospital	Network of Hospitals
Active patients	100	10,000	100,000
Average of sensors each one	10	6	4
Average time between updates	2	4	5
Number of users	5	20	100
List patients	45,000	180,000	900,000
List sensors	225,000	900,000	4,500,000
Recovery patient data	4,500,000	18,000,000	90,000,000
Recovery all values of a sensor (10% fails to cache)	1,125,000	4,500,000	22,500,000
Query of the last value of a sensor (8 hours/day, 5% fails to cache)	4,320,000	17,280,000	86,400,000
Update value of sensor	432,000,000	12,960,000,000	69,120,000,000
Monthly cost	$828.08	$24,653.12	$131,473.62
